# Advances in Bluetongue Virus Antigenicity and Vaccine Strategies: Progress and Perspectives

**DOI:** 10.3390/vetsci13101049

**Published:** 2026-10-07

**Authors:** Xinyi Li, Zhenghao Ye, Nijing Lei, Shaohua Pu, Huashan Yi, Xianping Ma

**Affiliations:** College of Veterinary Medicine, Southwest University, Rongchang, Chongqing 402460, China

**Keywords:** bluetongue, antigenic epitopes, inactivated vaccine, virus-like particle vaccine, DIVA (differentiation of infected from vaccinated animals)

## Abstract

Bluetongue (BT) is an infectious disease affecting domestic and wild ruminants caused by the Bluetongue virus (BTV). The development of safe and broad-spectrum vaccines represents the most effective approach for disease control. This review comprehensively elaborates recent advances in BTV research, including etiological characteristics, antigenic epitopes and immunity, inactivated vaccines (IVs), live attenuated vaccines (LAVs), virus-like particle vaccines (VLPs), viral vectored vaccines, subunit vaccines, disabled infectious single cycle (DISC) vaccines, and disabled infectious single animal (DISA) vaccines, providing valuable references for further investigations on BTV antigenic epitopes and vaccine development. Despite this progress, several critical limitations remain. Many next-generation vaccines improve safety and DIVA compatibility at the cost of reduced immunogenicity, and most novel platforms are evaluated mainly in mouse models with few field trials in natural target species. Moreover, owing to the serotype-specific nature of BTV, most vaccines still fail to provide broad-spectrum immunity. Future efforts should therefore prioritize target-species validation, conserved T-cell antigens such as NS1/NS2, and multivalent or chimeric vaccine design.

## Simple Summary

This review elucidates the antigenic architecture of Bluetongue virus (BTV) and the ensuing immune responses. It critically evaluates the advantages and disadvantages of conventional vaccines relative to emerging vaccine platforms. Furthermore, it underscores the imperative for future research to develop next-generation BTV vaccines that offer enhanced safety, broader cross-serotype protection and practicality.

## 1. Introduction

**Figure 1:**
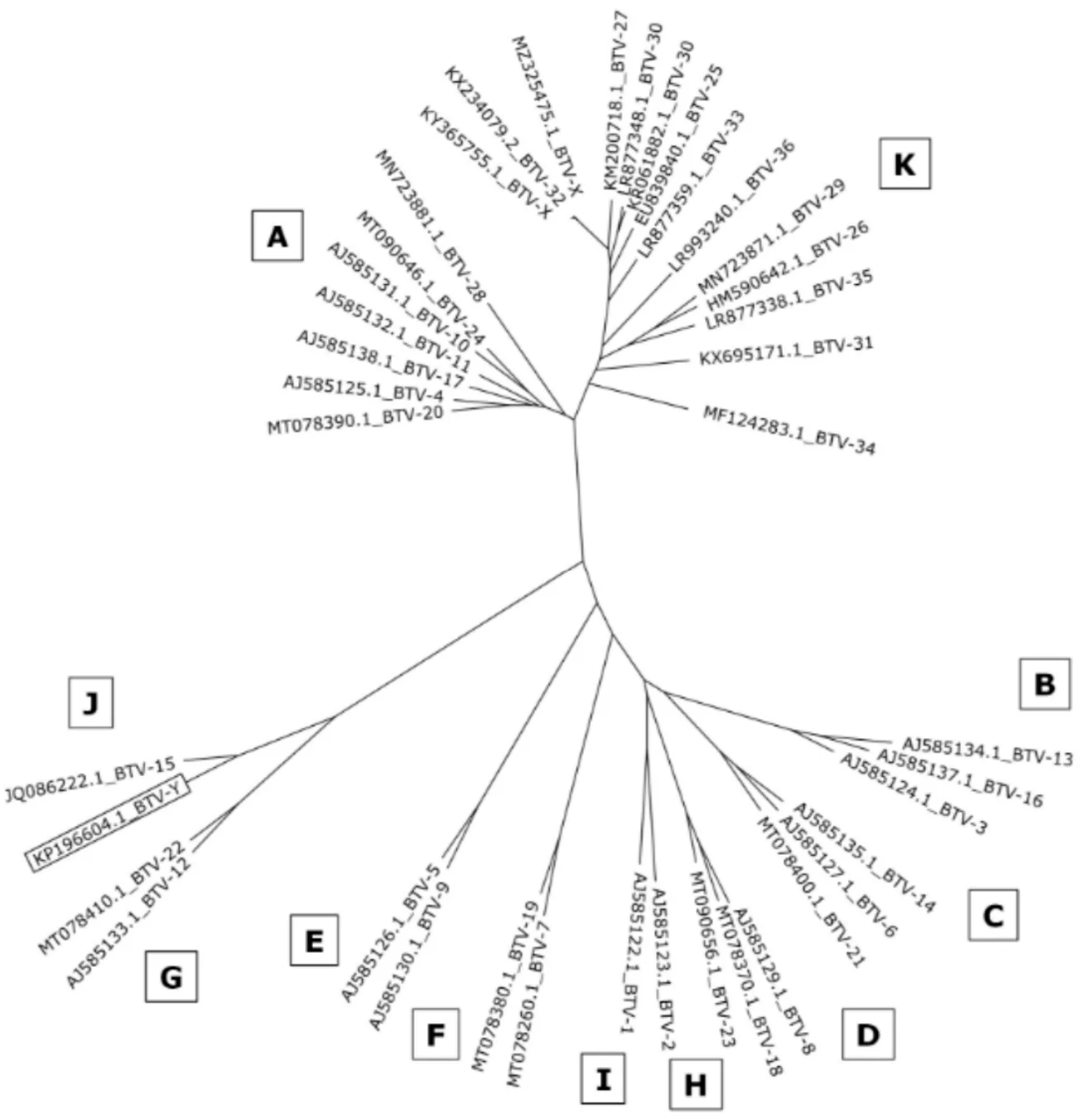
Seg-2 nucleotypes (NTs) are labeled A to K. BTV topotypes are omitted for clarity [3].

Bluetongue (BT) is a viral disease of domestic and wild ruminants caused by bluetongue virus (BTV) and transmitted by *Culicoides* spp. BTV is a dsRNA virus with 10 linear segments, classified in the genus Orbivirus of the family Sedoreoviridae [1]. BTV was first identified in 1876 in Merino sheep in the Cape Province of South Africa. In 1905, Arnold Theiler first confirmed that bluetongue was caused by a “filterable” pathogen, namely a virus. Then, Theiler developed the world’s first live attenuated BT vaccine based on the BTV-4 strain. It was not until the mid-20th century that the BTV VP2 serum neutralisation test (VNT) was successfully established. There is a constantly growing number of atypical serotypes with different viral characteristics that are genetically distinct from the 24 classical serotypes [2]. To date, at least 36 serotypes of BTV (BTV-1 to BTV-36) have been identified worldwide. BTV comprises 24 classical serotypes (BTV-1 to BTV-24) and an increasing number of atypical or proposed serotypes (BTV-25 to BTV-36). Recently, based on the sequence differences in Seg-2 among BTV serotypes 1 to 36, researchers classified them into 11 “nucleotypes” (NTs), designated A to K [3] (Figure 1). Sequence differences within the same NTs are less than 35%, while those between different NTs exceed 40% [4]. However, NT membership may provide a reference but requires cross-neutralization and target-animal validation.

**Figure 2:**
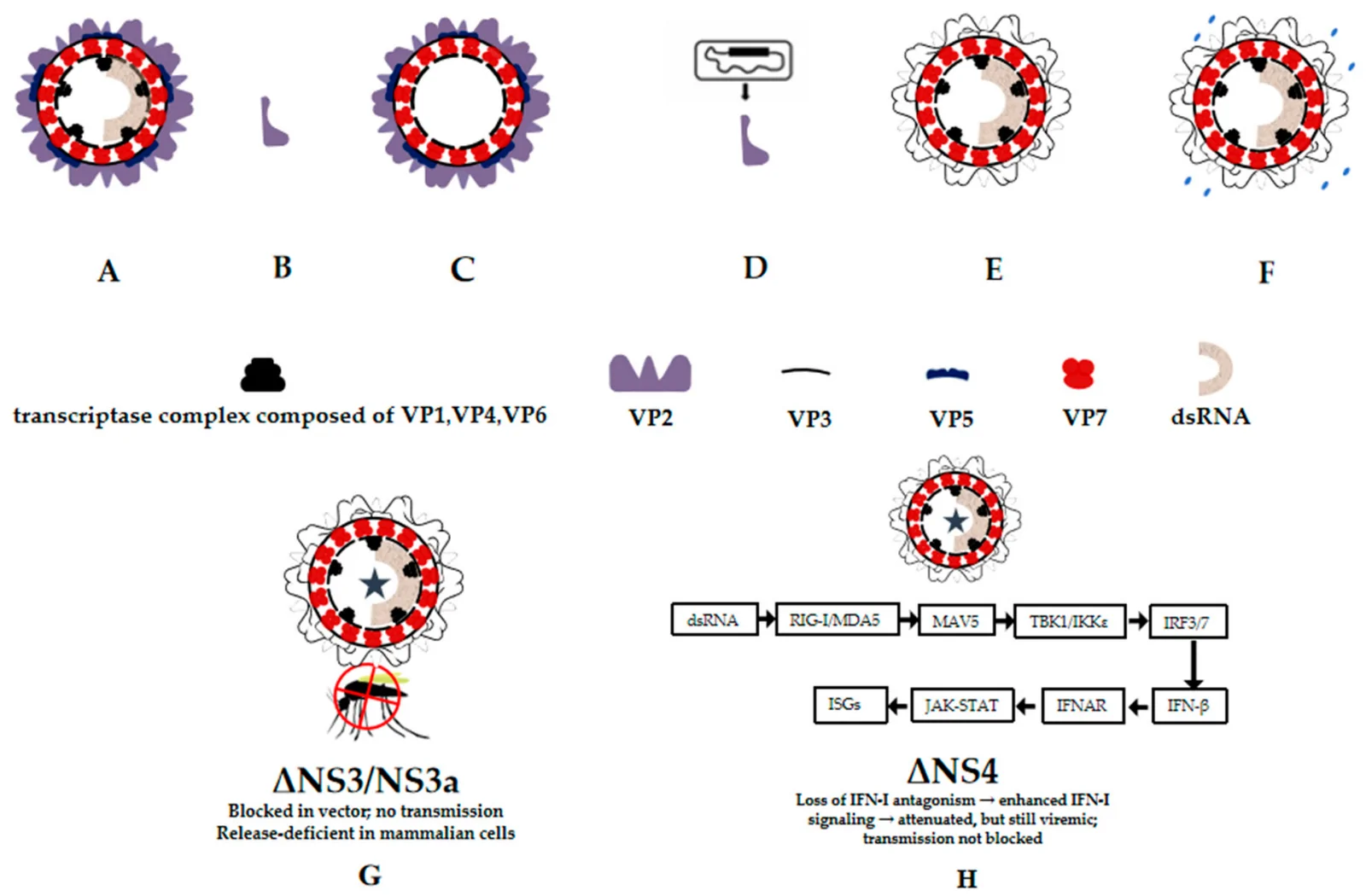
Overview of currently licensed and experimental BTV vaccine platforms. (**A**) Live-attenuated (LAV) or inactivated vaccine. (**B**) Subunit vaccine. (**C**) Virus-like particle (VLP) vaccine. (**D**) Recombinant viral-vectored vaccine. (**E**–**G**) Exchange of the serotype-determining outer-capsid proteins VP2 and VP5 (highlighted) between serotypes by reverse genetics. (**E**) “Serotyped” LAV and inactivated vaccines, or the production of a BTV backbone carrying the selected VP2/VP5. (**F**) DISC vaccine. (**G**) DISA vaccine, a replication-attenuated platform generated by deletion of NS3/NS3a (**H**) NS4-knockout vaccine. (Schematic concept adapted and modified from [6]).

Consequently, the low level of cross-protection between different serotypes poses a significant challenge to vaccine development. Over the past two decades, more than 5000 bluetongue outbreaks have occurred from 58° N to 35° S, causing economic losses of at least US $3 billion to the livestock industry [5] and threatening the health of domestic and wild ruminants worldwide. Safe and effective vaccines are regarded as the most effective means of preventing and controlling BTV infection. Countries have conducted extensive research into LAVs, IVs, subunit vaccines, VLP vaccines and viral vector vaccines for bluetongue (Figure 2) (Table 1).

Inactivated vaccines primarily induce humoral immunity, with CTL responses typically weak; whereas live-attenuated vaccines more closely mimic natural infection and can induce a certain degree of cellular immunity, although the magnitude and breadth vary depending on the vaccine strain, host, and assay method. Both conventional vaccine types, however, fail to allow differentiation between infected and vaccinated animals (DIVA) [6]. The immune response directed against non-structural proteins, together with CTL-mediated cellular immunity, is crucial for combating heterologous serotypes. Therefore, new vaccines are urgently needed to overcome these limitations. This review summarizes the pathogenic structure, antigenic characteristics and immune responses of BTV, and provides a systematic overview of both conventional and novel BT vaccines to aid future vaccine development. We conducted a comprehensive search of PubMed, Web of Science, Scopus, CNKI and NCBI for studies on BTV antigenicity and vaccines published up to June 2026, using terms such as “bluetongue virus”, “vaccine”, “antigenic epitope”, and “DIVA”. Peer-reviewed original research, clinical trials, and authoritative reviews were included, whereas duplicate, non-peer-reviewed, and irrelevant records were excluded. Ultimately, 80 references were selected for inclusion.

## 2. Pathogenic Characteristics of BTV

### 2.1. The Structure and Life Cycle of Viruses

BTV particles are non-enveloped and icosahedral. Their genome encodes seven structural proteins (VP1–VP4 from Seg1–Seg4, VP5 from Seg6, VP6 from Seg9, VP7 from Seg7) and at least six non-structural proteins (NS1 from Seg5, NS2 from Seg8, NS3/NS3a from Seg10, NS4 from Seg9, NS5 from Seg10) [29]. VP2 and VP5 together form the outer capsid of BTV. The remaining proteins, together with the dsRNA segments, form the viral core particle. VP1 (RNA polymerase), VP4 (capping enzyme) and VP6 (RNA helicase) are three minor proteins with enzymatic activity. Within the BTV virion, they form the viral transcriptase complex (TC). Together with dsRNA segments, they also constitute the core-like particles (CLPs), which are encapsidated by VP3 [30].

**Figure 3:**
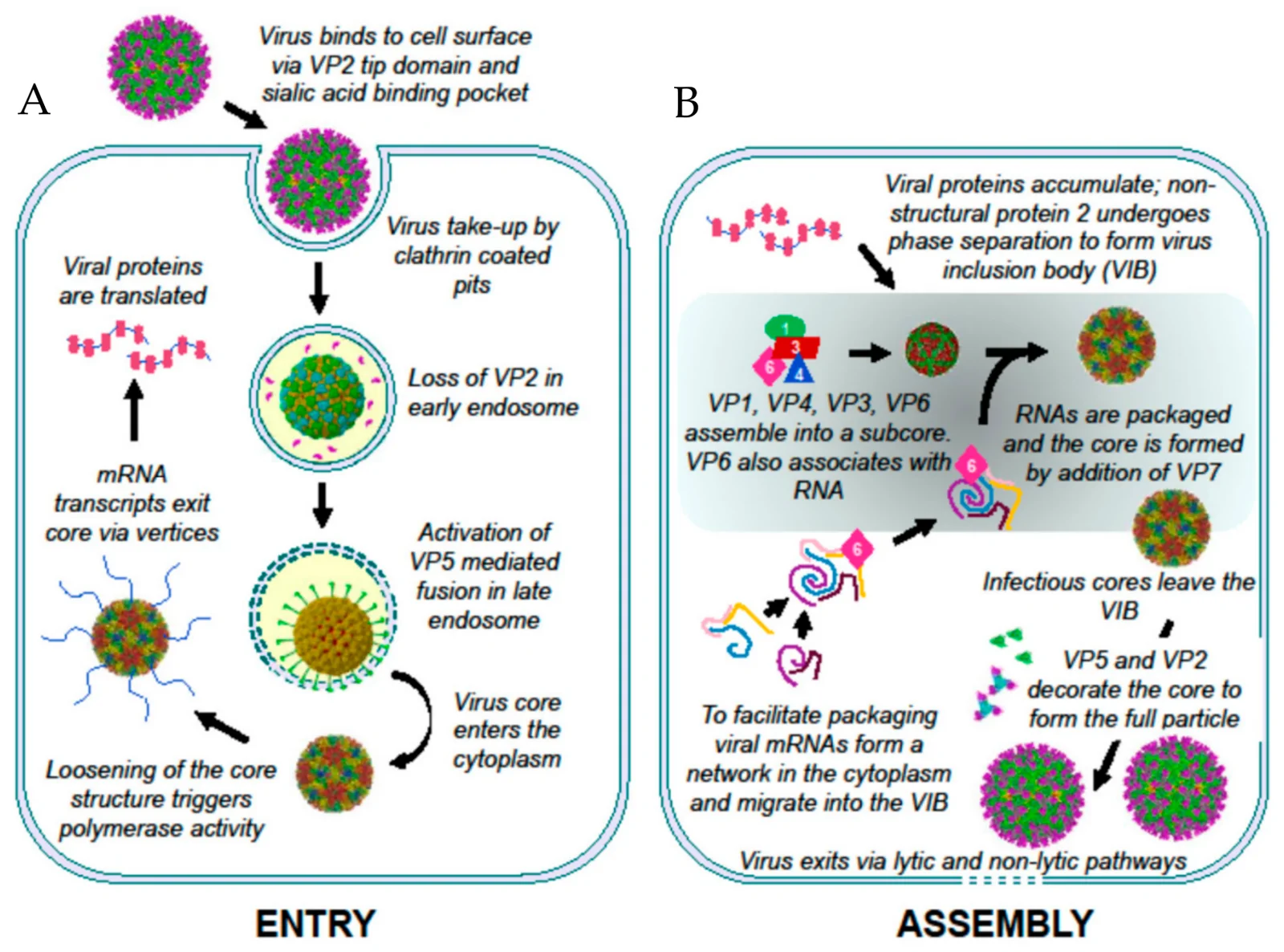
Schematic diagrams of Virus Entry and Assembly [36]. (**A**) BTV attaches to and enters cells via clathrin-mediated endocytosis (CME). Structural and non-structural proteins (VPs/NSPs) are synthesized intracellularly. (**B**) Assembly and release of daughter virus particles. The numbers in the figure have already indicated that they are respectively VP1, VP3VP4 and VP6.

The BTV life cycle begins with the VAYTLKPTY motif within the VP2 to α2,3- and α2,6-sialic acid receptors on the host cell, followed by internalisation into an early endosome (EE) where VP2 is degraded. This triggers a conformational shift in N-terminal dagger domain (DD) of VP5, allowing its M1-S41 and WHXL (W411-L414) motifs to promote endosomal membrane fusion (Figure 3A). Upon complete degradation of VP2, the DD and unfurling domains (UD) of VP5 refold and localise to late endosomes (LEs). Finally, transcriptionally active CLPs are released from the late endosomes [30]. Meanwhile, NS4 and NS5 antagonize interferon-mediated immune responses to evade host immunity [31]. Activated VP1 and VP6 unwind dsRNA and transcribe positive-sense ssRNA, which is capped by VP4 and then released into the cytoplasm through the VP7 pentamer [32]. This capped ssRNA subsequently serves as both a replication template and mRNA for the synthesis of viral structural proteins (VP1-VP7) and non-structural proteins (NS1-NS4) [32]. Studies have shown that the BTV NS1 protein, in its non-tubular monomeric form, specifically recognizes and binds to the 3’terminal ACUUAC hexanucleotide of viral single-stranded RNA, thereby upregulating the translation efficiency of viral mRNA and promoting the preferential synthesis of viral proteins. Meanwhile, NS1 monomers can polymerize to form tubular structures. These tubular structures depend on the cellular microtubule network for intracellular transport and ultimately aggregate at the microtubule organizing center, where they are encapsulated by vimentin to form aggresomes [33]. NS2 is the major component of viral inclusion bodies (VIBs), serving as the replication site and enhances assembly efficiency. In addition, both NS1 and NS2 affect the mitosis of host cells [29,31,34]. VIBs recruit newly synthesized proteins (VP1, VP4, VP6, VP3, VP7) and ssRNA to assemble CLPs. Some of the newly assembled daughter CLPs replicate and proliferate within host cells, just like their parent CLPs (Figure 3A). The remaining CLPs leave the VIBs after acquiring VP5 within them. They acquire VP2 in the cytoplasm, form complete viral particles, and diffuse out of the cell (Figure 3B) [35].

### 2.2. Antigenic Epitopes of Capsid Proteins and Humoral Immunity

The BTV-specific outer capsid proteins VP2 and VP5 are critical for viral entry into host cells and also contain the primary epitopes that induce humoral immunity. VP5 acts as a membrane-penetrating protein, while VP2 acts as a receptor-binding protein [37]. VP2 and VP5 each form homotrimers, which together constitute the outer capsid protruding from the viral surface. Structural dissection of VP2 has revealed four distinct subdomains: the hub domain (HD), the hairpin domain (HpD), the body domain (BD) and the tip domain (TD) [38,39,40]. In particular, TD not only mediates viral attachment and entry but also serves as a key site for inducing humoral immunity. Following infection with BTV, the host cell receptor binds to the TD of VP2. The virus is taken up by dendritic cells (DCs) in animal tissues or skin and transported to lymph nodes. Ultimately, antigenic epitope peptides are presented to lymphocytes, thereby triggering an adaptive immune response [31].

Specific neutralising antibodies produced by activated B cells can bind to VP2. They neutralise the virus by blocking the interaction between BTV VP2 and α2,3-sialic acid receptors on the surface of host cells [1,41]. Therefore, the full-length VP2 protein or its key peptides are often used as the core components of novel vaccines (Table 2). Studies have shown that the VP5 protein also contains antigenic epitopes that can be recognized by host B cells (Table 3) [42,43,44]. However, the VP5 antigenic epitopes that have been analyzed are primarily linear peptides [43,45]. To date, their three-dimensional structures have not been precisely determined. It should be noted that although the VP2 epitopes listed in Table 2 are predominantly linear, the dominant neutralization determinant of VP2 is conformational and serotype-specific [1]. Antibodies raised against denatured VP2 or synthetic peptides often fail to neutralize the virus, whereas antibodies against intact virions exhibit neutralizing activity. This methodological bias arises because linear epitope mapping via peptide scanning is technically more accessible than the structural resolution of antigen–antibody complexes. Consequently, linear epitopes are more suitable for subunit and multi-epitope vaccine design due to their stability and ease of synthesis, whereas conformational epitopes represent the authentic targets of protective neutralizing antibodies and should be prioritized for structure-based vaccine design. Although VP5 is less immunogenic than VP2, co-immunization with VP2 significantly enhances the neutralising antibody response. Therefore, in the preparation of BTV VLP vaccines, mRNA vaccines, or recombinant vector vaccines, VP2 and VP5 proteins (or structural multimers containing these proteins) are used as core antigens in order to elicit high-titer neutralising antibodies. On the other hand, Bissett’s team found that the TD mutation can lead to a loss of antibody recognition [1], and VP2 exhibits a high degree of resistance to mutations in this domain. The virus can evade recognition by neutralising antibodies through minor mutations in the TD. Due to mutations in the TD, there is significant variation in the VP2 TD among different serotypes, making it difficult for neutralising antibodies to provide cross-protection.

### 2.3. Non-Structural Proteins and Cellular Immunity

BTV non-structural proteins (NS1, NS2, NS3, and NS4) play a key role in inducing cellular immunity. The N-terminus of BTV NS1 contains a 9-amino acid peptide (NS1-152). It has been identified as a key antigenic epitope that mediates cytotoxic T lymphocyte (CTL) responses [59]. Andrew [60] discovered that NS1 in sheep is the primary immunogen for CTLs. NS1-specific CTLs cross-react with cells infected with different BTV serotypes and mediate CD8+ T-cell responses. Therefore, BTV NS1 is considered a potential broad-spectrum candidate antigen for cellular immunity due to its high conservation across serotypes and its cross-protective properties observed mainly in IFNAR−/− mice [59]. However, heterologous challenge validation in sheep and cattle is still required.

Andrew [60] further confirmed, through the reorganization of the vaccinia virus system, that BTV-specific CTLs in sheep primarily recognize NS1 (the major immunogen) and NS3 (the minor immunogen). Kolla [61] identified multiple 100% conserved CD4+ T-cell epitopes in NS2 across 24 serotypes. In contrast, both CD8+ and CD4+ T-cell epitopes were present in NS1 and NS3. On the other hand, Anderson confirmed that BTV NS1 and NS2 can induce specific lymphocyte proliferation and antibody responses. However, NS3 has a weaker ability to induce cellular immunity [15]. NS1 is a key inducer of cross-reactive CTLs in mouse models, while NS2 is a key protein in inducing a conserved CD4+ T-cell response. The combination of these two approaches may provide a basis for the development of a broad-spectrum, multi-epitope vaccine, but this remains to be validated in target species Although NS3 has low immunogenicity, its high conservation makes it a suitable negative marker in the DIVA strategy.

## 3. BTV Vaccines

### 3.1. Attenuated Live Vaccines and Inactivated Vaccines

LAVs and IVs are the two most commonly used types of vaccines in BTV vaccine research and application (Figure 2A). In 1906, Theiler used blood from sheep naturally infected with BTV as the vaccine strain and attenuated it through successive passages in sheep. The resulting blood was used to develop the first monovalent BTV-4 LAV [7,62]. Various combinations of the Onderstepoort LAV freeze-dried bovine vaccine developed by Dungu et al. are widely used in South Africa. The Onderstepoort LAV consists of Vial A (BTV-1, 4, 6, 12, and 14), Vial B (BTV-3, 8, 9, 10, and 11), and Vial C (BTV-2, 5, 7, 13, and 17) [7]. With the exception of South Africa, countries are developing vaccines against specific BTV serotypes circulating in their regions [8,9]. Examples include the LAVs of BTV-10, BTV-11, and BTV-17 in the United States; the LAVs of BTV-1 and BTV-16 in China; and the LAV of BTV-4 in Turkey and Morocco. However, the use of BTV LAVs has been associated with several adverse effects in target animals, including pyrexia, reproductive failures, diminished milk yield, and impaired semen quality in rams [63]. Second, LAVs not only pose a risk of genetic recombination between their gene segments and those of wild-type strains, but it may also serve as a potential source of transmission through vectors such as midges [64,65].Vaccine strains of attenuated live BTV vaccines can be transmitted from immunized to unvaccinated animals via *Culicoides* midges, and their vertical transmission capacity is not only retained but occasionally enhanced. Although the well-established Onderstepoort Biological Products bluetongue (BT) vaccine has a long history of safe use, with no documented reversion to virulence or vector-borne transmission [8], attenuated live BTV vaccines, in general, should therefore still be regarded as carrying a theoretical risk of reversion to virulence and vector transmission [64], and their safety needs to be assessed on a case-by-case basis according to vaccine strain, serotype, host, and region.

Studies have shown that BTV IV is safer. There is no risk of virulence resurgence or genetic reassortment [66]. Therefore, BTV IV remains an important conventional tool for controlling BTV in most BTV-free countries to this day. The European BTV-8 epidemic is a well-documented example of rapid vaccine development and successful application. BTV-8 emerged in north-western Europe in 2006, and large-scale vaccination with inactivated BTV-8 vaccines was implemented principally in 2008, which markedly reduced clinical disease and virus circulation [10]. February 2025, the European Commission granted marketing authorisations under exceptional circumstances for three inactivated BTV-3 vaccines: BLUEVAC-3, Syvazul BTV 3, Bultavo 3 (Table 1). Field data from Dutch dairy cattle herds demonstrated that herds fully vaccinated with Bultavo 3 showed a significant reduction in the probability of detecting BTV-3 RNA compared to non-vaccinated herds [11]. In Belgian herds, seropositivity rates were 89.7% for Bluevac-3, 90.0% for Syvazul BTV-3, and 74.8–100% for Bultavo-3 depending on the number of doses administered [67]. In addition, a candidate inactivated BTV-4 vaccine induced the onset of immunity at the latest at day 21 after the first dose in more than 80% of vaccinated individuals, with specific antibodies detected for more than one year. Challenge experiments indicated that the vaccine protected a high percentage of sheep or significantly reduced the intensity and duration of clinical signs [68]. Of note, however, the neutralising antibody levels measured by conventional serological assays are often low [69], and in some cases even fall below the detectable threshold—despite vaccinated animals being effectively protected from clinical infection. This apparent paradox highlights a critical limitation of IVs, which primarily induce humoral immunity with weak CTL responses. LAVs can induce robust cellular immunity, but their safety concerns limit their use. Given these inherent limitations, there is an urgent need to develop next-generation vaccines that overcome these shortcomings.

### 3.2. Subunit & Epitope-Based Vaccines

Notably, the first BTV subunit vaccine to reach regulatory approval has recently been developed: VeroBlue-3, a recombinant vaccine based on the BTV-3 VP2 protein. Following a positive opinion adopted by the EMA Committee for Veterinary Medicinal Products (CVMP) in March 2026, VeroBlue-3 was granted an EU marketing authorisation under exceptional circumstances in April 2026 for active immunisation of sheep to reduce viraemia and pyrexia caused by BTV-3, with onset of immunity 14 days after primary vaccination [12]. Legisa et al. [14] expressed the VP2 protein of an Argentine BTV-4 isolate in a baculovirus-insect cell expression system. The expressed products included VP2 alone and a fusion protein designated APCH-VP2. The APCH-VP2 fusion protein contains an antigen-presenting cell homing (APCH) molecule. In this way, they developed an experimental subunit vaccine capable of targeting recombinant proteins to antigen-presenting cells. The vaccine induces high titers of specific neutralising antibodies in guinea pigs and cattle, at levels comparable to those of conventional inactivated vaccines [14]. Anderson et al. used AbISCO-100 as an adjuvant and expressed BTV-8 VP2 and VP5, as well as BTV-1 NS1, NS2, and NS3 proteins, using baculovirus systems and E. coli systems. They constructed a novel subunit vaccine targeting BTV-8 that allows differentiation of infected from vaccinated animals. The experimental vaccine containing BTV-8 VP2, BTV-2 NS2 and BTV-2 NS1 was evaluated in challenge experiments [15]. The vaccine induced robust neutralising antibodies against BTV-8 and cross-reactive T-cell responses against the NS1/NS2 proteins, which are likely important for protection. Furthermore, the ISCOM-based adjuvant AbISCO-100 facilitates the transport of antigens to major histocompatibility complex class I (MHC-I) and class II (MHC-II) molecules via antigen-presenting cells (APCs), then inducing an immune response in CD8+ T cells and CD4+ T cells. T-cell epitopes (MHC-I/II) that are highly conserved across 24 serotypes were screened from the NS1 and NS2 proteins of BTV by Kolla et al. [61] using immunoinformatics methods. Based on these epitopes, a broad-spectrum multi-epitope subunit vaccine containing a Toll-like receptor 4 (TLR4) agonist was computationally designed, and it was predicted to bind the TLR4 receptor and to induce the type 1 helper T-cell (Th1) cellular immune response. Jyothi et al. [70] found that although BTV VP2 exhibits high overall variability, the amino acid region spanning positions 338–383 (the conserved region of VP2, cVP2) is relatively conserved and contains multiple B-cell and T-cell (MHC-I/II) epitopes. Therefore, Jyothi predicted that cVP2 may serve as a candidate target for a broad-spectrum subunit vaccine. An in silico multi-epitope subunit vaccine was designed by Sinha et al. [26] against six serotypes of BTV (4, 10, 11, 17, 20, and 24). The vaccine included B cell epitopes, CD4+T-cell epitopes, and CD8+T-cell epitopes. Numerous studies have shown that the immune effect of two or more antigens is superior to that of a single-antigen vaccine. Through antigen screening, APCH-targeted delivery, and multi-epitope design, the immunogenicity and broad-spectrum efficacy of the vaccine can be further enhanced (Figure 2B).

### 3.3. Virus-like Particle (VLP) Vaccines

VLPs are hollow particles self-assembled from one or more viral structural proteins. They resemble native viruses in appearance but contain no genetic material, making them non-infectious and replication-defective (Figure 2C). VLPs have a particle size between 20 and 200 nm. Compared to BTV subunit vaccines, BTV VLP vaccines are more easily recognized and captured by APCs. Furthermore, the highly repetitive antigen arrangement on the surface of VLPs can be used to efficiently activate the immune system, and high titers of neutralising antibodies are induced. Therefore, both safety and good immunogenicity are exhibited by VLP vaccines [71]. BTV VLP vaccines are typically expressed using baculovirus-insect cell and plant expression systems. The four structural proteins VP2, VP3, VP5, and VP7 are expressed by these systems. These proteins then self-assemble into BTV VLP vaccines in the heterologous systems. A baculovirus vector carrying the polyhedrin promoter was used by Roy et al. to co-express VP3 and VP7 in insect cells. BTV-16 core-like particles (CLPs) were successfully assembled. Furthermore, the four structural proteins (VP2, VP3, VP5, and VP7) were co-expressed, and BTV VLP vaccines were subsequently assembled [72,73]. The baculovirus-insect cell system can efficiently express correctly folded recombinant proteins. It is also safe for vertebrates. However, this system produces a large number of infectious baculovirus particles, which are difficult to separate from VLP vaccines and thus interfere with vaccine immunogenicity [74,75]. To address this limitation, a recombinant baculovirus system with a deletion of the vp80 gene was developed by Marek’s team. Through trans-complementation, BTV VLP vaccines containing recombinant proteins but free of baculovirus particles can be produced by this system [76]. Based on the Agrobacterium-mediated Nicotiana benthamiana transient expression system first reported by Van Zyl’s team [77], chimeric VLP vaccines were successfully assembled by Gwynn through co-expression of the chimeric BTV1/8 VP2 protein together with the BTV-8 VP3, VP5, and VP7 proteins. High-level neutralisation of both BTV-1 and BTV-8 serotypes was achieved simultaneously by these chimeric VLP vaccines. The baculovirus expression system is mature, but contamination issues are present. Lower cost and higher safety are achieved by the plant transient expression system. In the future, the contamination problem of the baculovirus system needs to be resolved, and the industrial application of the plant system should be promoted. By doing so, the safety and feasibility of BTV VLP vaccines can be improved.

### 3.4. Viral Vector Vaccines and Combination Strategies

Adenovirus, herpesvirus, and poxvirus vectors are often used for BTV viral vector vaccines (Figure 2D). These vectors can easily insert large fragments of foreign genes and induce strong cellular and humoral immune responses. DIVA is also facilitated by such vaccines [19,21,78,79]. In 2015, Feng Yufei’s team developed a BTV recombinant adenovirus vaccine, rAd-VP2, and rAd-VP2-IRES-VP5, based on an adenovirus vector platform [16]. The team of Martín V performed co-immunization with a recombinant replication-defective Ad5VP7-8 and Ad5VP2-1. Cross-protection against BTV-1, BTV-8, and heterologous BTV-4 was conferred by this regimen [18,80]. Ma et al. inserted the VP2 and VP5 genes of BTV-8 into the bacterial artificial chromosome (BAC) of the attenuated strain RacH of equine herpesvirus type 1 (EHV-1), then successfully constructed a recombinant viral vaccine named rH_EF1_VP2_5 [19]. Li Junping’s team constructed a universal transfer vector designated pSY681-gfp-gpt. They employed a prime-boost immunization strategy against the Chinese epidemic strains of BTV-1 and BTV-16. High levels of neutralising antibodies were successfully induced by this approach [20].

Due to multiple deletions in its genome, the modified vaccinia virus Ankara (MVA) serves as an important vaccine vector platform. Jiménez-Cabello et al. recently demonstrated that co-expression of VP2, NS1, and NS2-Nt proteins by an MVA viral vector induced complete protection against BTV-4M challenge in sheep [21]. This represents a paradigm shift in BTV vector vaccine design from “neutralizing antibody-dependent” to “combining neutralizing antibodies and T-cell immunity.” By moving beyond VP2 as a single target and incorporating the conserved NS1/NS2 proteins, this strategy induces cross-reactive T-cell immunity, providing serotype-specific protection while broadening the protective spectrum and circumventing the immune imprinting limitation of VP2. Lobato et al. used the Western Reserve (WR) strain of vaccinia virus as a vector to construct recombinant viruses expressing BTV-1 VP2, VP5, or both VP2 and VP5. The most durable protection was provided by co-immunization with VP2 and VP5, as demonstrated by the challenge experiment [81]. Jabbar et al. cloned the VP2, VP5 and VP7 genes of BTV-8 into the pCI-neo mammalian expression vector to prepare a DNA vaccine. In IFNAR−/− mice, this vaccine induced neutralising antibodies against BTV-8, and DNA/rMVA or rMVA/rMVA prime-boost provided complete protection against lethal BTV-8 challenge in this mouse model [82]. In conclusion, cellular immunity can be used to target antigenic determinants shared by multiple BTV serotypes. Overall, the combined immunization strategy of a DNA vaccine and other viral vector vaccines is also investigated. Both of these approaches contribute to the development of multiserotype BTV vaccines.

### 3.5. Disabled Infectious Single Cycle (Disc) Vaccines and Disabled Infectious Single Animal (Disa) Vaccines

DISA and DISC are two next-generation BTV vaccine platforms, both based on reverse genetics (Figure 2F,G). The DISC vaccine is generated by deleting the VP6 gene, which encodes a minor core protein essential for viral transcription and core assembly [32]. In vaccinated animals, the DISC virus can enter cells and complete a single round of infection—it is therefore “entry-competent.” However, because the viral genome lacks VP6, newly synthesized viral proteins cannot assemble into infectious core particles, and no progeny virus is produced. This principle is termed “entry-competent replication-abortive” (ECRA) [83]. Matsuo et al. first generated a VP6-deficient replication-defective BTV-8 DISC vaccine virus using a reverse genetics system, based on the BS9 complementing cell line that provided the VP6 protein in trans. Subsequently, the team replaced the two RNA segments encoding the outer capsid proteins VP2 and VP5 from a highly virulent BTV-1 strain with those of the BTV-8 DISC backbone. Through this reassortment approach, they successfully constructed a bivalent recombinant DISC vaccine candidate designated BTV1/8D1 [23]. For DIVA, naturally infected animals develop antibodies against VP6, whereas DISC-vaccinated animals do not, allowing differentiation by a VP6-specific ELISA. Based on the studies of Matsuo, Celma constructed a DIVA vaccine targeting six serotypes: BTV-1, BTV-2, BTV-4, BTV-8, BTV-13, and BTV-21 [25].

The DISA vaccine is a live-attenuated BTV from which the non-essential NS3/NS3a proteins have been deleted. As a result, it cannot replicate in the vector insect (*Culicoides* midges). For DIVA, naturally infected animals produce antibodies against NS3, whereas DISA-vaccinated animals do not because the vaccine virus does not express NS3/NS3a. A companion NS3-specific competitive ELISA allows clear differentiation between infected and vaccinated animals [24,28]. Van Rijn et al. developed a DISA/DIVA platform based on the BTV-6 backbone. This platform introduced a 72-aa deletion mutation into genome Seg-10. Using the resulting LAV strain BTV-6/net08, they further constructed the DISA8 vaccine. A large body of work has shown that swapping the VP2- and VP5-coding genes across different BTV serotypes can produce monovalent or multivalent DISA vaccines that target the corresponding serotypes. Using either heterologous combinations or chimeric Seg-2 and Seg-5 designs, researchers have also successfully developed DISA vaccines covering eight serotypes: BTV-1, BTV-2, BTV-3, BTV-4, BTV-6, BTV-8, BTV-17, and BTV-25 [24,27]. Among these, a pentavalent formulation (BTV-1, 2, 4, 6, and 8) has been shown to provide protection in sheep and cattle [27]. Fundamentally, DISC and DISA differ in their replication dynamics within the vaccinated host. DISC exhibits abortive replication due to VP6 deficiency, whereas DISA is release-deficient and vector-incompetent owing to the deletion of NS3/NS3a. Consequently, their respective DIVA markers are VP6 for DISC and NS3 for DISA. Nevertheless, further work is needed in several areas, including tissue tropism and immune responses, multivalent and multi-pathogen gene-deletion technologies, nano-adjuvants, needle-free immunization methods, the maintenance of immune memory, and cross-species adaptability.

## 4. Discussion

Because the dominant neutralization determinants of VP2 are conformational and serotype-specific, whereas most mapped epitopes are linear, future vaccine designs should combine linear epitopes for multi-epitope subunit vaccines with structure-based strategies targeting conserved conformational epitopes. The synergistic effect of VP2 and VP5 co-immunization, together with the conserved T-cell epitopes in NS1, supports the inclusion of these antigens in next-generation vaccines [1,5,21,61,84]. However, the cross-protective potential of NS1 still requires validation in genetically heterogeneous sheep and cattle.

From a veterinary practice perspective, each BTV vaccine platform shows a distinct profile of practical advantages and limitations. LAVs offer strong, durable, low-cost, single-dose immunity but raise safety, reversion, vector-transmission and DIVA-incompatibility concerns. IVs are safer and reduce clinical disease, yet require boosters, induce weak CTL responses, and show inconsistent viraemia blocking. Subunit and VLP vaccines are DIVA compatible, but subunit immunogenicity is weak, VLP manufacturing needs optimization, and target-species viraemia data are limited. Viral vector vaccines induce cellular and humoral immunity but often require prime-boost and are constrained by VP2 immune imprinting. DISC and DISA provide DIVA and transmission control: DISC is replication-abortive, while DISA is vector-incompetent and cannot replicate in *Culicoides*, a unique advantage. However, most new vaccines still lack long-term field data on clinical protection, viraemia reduction, duration of immunity, and single-dose feasibility. Future studies should therefore prioritize these practice-oriented endpoints.

In summary, the core bottlenecks in current BTV vaccine development are concentrated in three aspects: first, the high variability of VP2 leads to insufficient cross-protection, and a single point mutation in the tip domain (TD) can mediate escape from neutralizing antibodies; second, cellular immunity induced by existing vaccines is generally insufficient to counter heterologous serotypes; third, most novel vaccines achieve DIVA compatibility at the cost of partial immunogenicity, making it difficult to simultaneously achieve “safety/DIVA compatibility” and “immune efficacy”. To address these bottlenecks, future breakthrough paths should focus on designing broad-spectrum multi-epitope vaccines based on conserved T-cell epitopes such as NS1/NS2, constructing chimeric VLPs or optimizing multivalent DISA vaccine systems using reverse genetics, and developing novel adjuvants and delivery systems to enhance immune responses.

## 5. Conclusions

In conclusion, BTV vaccinology is undergoing a decisive paradigm shift from conventional inactivated and live-attenuated vaccines toward next-generation multivalent and vector-based platforms. Recent innovations—ranging from plant-derived chimeric virus-like particles (VLPs) and reverse genetics-engineered synthetic strains to MVA-vectored candidates and NS3/NS3a-deleted DISA/DIVA vaccines—exemplify this transition. For DNA–adenovirus prime-boost regimens, DIVA compatibility depends on the absence of NS3/NS3a from the vaccine and the use of a validated NS3-specific ELISA; this remains to be confirmed in sheep and cattle. Collectively, these advanced strategies not only enhance biosafety and broaden protective efficacy but also enable critical serological differentiation between vaccinated and naturally infected animals, thereby overcoming the longstanding limitations of traditional prophylactic approaches.

## 6. Future Directions

To achieve safe and broad-spectrum BT control, future progress will depend less on introducing new technologies and more on rigorously validating the platforms already supported by animal data, particularly through target-species challenge studies, long-term field trials, and standardized DIVA diagnostics. Yet a critical concern is that multivalent formulations may dilute the immune response to individual serotypes, and no long-term field data exist to confirm whether cross-protection is maintained under natural challenge. Although mRNA vaccine technology remains at an early stage for BTV with no peer-reviewed animal data, it represents a potential long-term direction once conserved neutralizing epitopes are identified. In summary, future progress will depend less on introducing new technologies and more on rigorously validating the platforms already supported by animal data.

## Figures and Tables

**Table 1:** Selected BTV vaccine platforms with regulatory approval or advanced development status.

Vaccine Name (Type)	Serotype(s)	Developer (Country)	Regulatory Status	Evidence Level
OBP 15-valent LAV (live attenuated) [7,8]	15 serotypes	Onderstepoort Biological Products (South Africa)	National authorization (South Africa)	Licensed/field data
BTV-10/11/17 LAV (live attenuated) [8]	BTV-10, 11,17	Manufacturer not specified (USA)	National authorization (USA)	Target species field data
BTV-1/16 LAV (live attenuated) [8]	BTV-1, 16	Lanzhou Veterinary Research Institute, CAAS (China)	National authorization (China)	Target species field data
BTV-4 LAV (live attenuated) [8]	BTV-4	Manufacturer not specified (Turkey/Morocco)	National authorization	Target species field data
BTVPUR AlSap 8 [9] (inactivated)	BTV-8	Merial S.A.S./Boehringer Ingelheim (France)	EU centralized authorization (2009)	Target species field data
Zulvac^®^ 8 (inactivated) [9]	BTV-8	Zoetis Belgium SA (Belgium)	EU centralized authorization (2009)	Target species field data
BLUEVAC^®^ 8 (inactivated) [10]	BTV-8	CZ Veterinaria/CZ Vaccines (Spain)	EU centralized authorization (2009)	Target species field data
BTV-1/16 inactivated vaccine (inactivated) [8]	BTV-1, BTV-16	Lanzhou Veterinary Research Institute, CAAS (China)	National authorization (China)	Target species field data
BTV-2/18 monovalent and BTV-1/2/15/18/23 [8] pentavalent (inactivated)	BTV-2/18; BTV-1/2/15/18/23	Indian Veterinary Research Institute (India)	National authorization/trial	Target species experimental data
BLUEVAC-3 (inactivated) [11].	BTV-3	CZ Vaccines S.A.U. (Spain)	EU centralized authorization, exceptional circumstances (Feb 2025)	Licensed/field data (target species); sheep and cattle
Syvazul BTV 3 (inactivated) [11].	BTV-3	Laboratorios Syva S.A. (Spain)	EU centralized authorization, exceptional circumstances (Feb 2025); sheep	Licensed/field data (target species); sheep
Bultavo 3 (inactivated) [11].	BTV-3	Boehringer Ingelheim Vetmedica GmbH (Germany)	EU decentralized authorization (April 2025)	Licensed/field data (target species)
VeroBlue-3 [12] (inactivated viral vaccine)	BTV-3	VERVACCINES GmbH (Germany)	EU marketing authorisation in exceptional circumstances	Licensed; EMA-assessed efficacy; sheep
BTV-16 VP2/VP5-41 subunit vaccine [13] (subunit)	BTV-16	CAAS (China)	Experimental	Target species experimental data
VP2-APCH fusion subunit vaccine (subunit) [14]	BTV-4	INTA (Argentina)	Experimental	Target species (cattle, guinea pigs, IFNAR-/- mice)
Multi-serotype subunit vaccine [15] (subunit)	BTV-2, BTV-8	Statens Serum Institut/University of Copenhagen (Denmark)	Experimental	Target species experimental data
rAd-VP2, rAd-VP2-IRES-VP5 (viral vector) [16]	BTV-16	Northeast Agricultural University (China)	Experimental	Mouse/target species
Canarypox VP2/VP5 vaccine [17] (viral vector)	BTV-17	Sanofi Pasteur/Merial (USA)	Experimental	Target species (sheep)
Ad5VP7-8 + Ad5VP2-1 (viral vector) [18]	BTV-1, BTV-8, BTV-4	CISA-INIA (Spain)	Experimental	Target species (sheep)
rH_EF1_VP2_5 [19] (viral vector)	BTV-8	Freie Universität Berlin/FLI (Germany)	Experimental	Mouse model only
DNA prime/FPV boost [20] (viral vector)	BTV-1, BTV-16	Lanzhou Veterinary Research Institute, CAAS (China)	Experimental	Target species
MVA-VP2-NS1-NS2-Nt [21] (viral vector)	BTV-4	CISA-INIA (Spain)	Experimental	Target species (sheep)
BTV-8 VLP vaccine (VLP) [22]	BTV-8; BTV-1, 2, 8 (cocktail)	INRA/ANSES (France)	Experimental	Target species (sheep)
BTV1/8D1 DISC [23] (replication-defective)	BTV-1, BTV-8	LSHTM/ANSES (UK/France)	Experimental	Target species (sheep)
DISA8 (replication-deficient) [24]	BTV-8	Wageningen Bioveterinary Research (Netherlands)	Experimental	Target species (sheep)
Hexavalent DISC vaccine (replication-defective) [25]	BTV-1, 2, 4, 8, 13, 21	The Pirbright Institute (UK)/international consortium	Experimental	Target species (sheep, cattle) challenge protection
Multi-epitope subunit vaccine (subunit) [26]	BTV-4, 10, 11, 17, 20, 24	ICAR-NIVEDI/IBAB (India)	Experimental	In silico only
Pentavalent DISA (replication-deficient) [27]	BTV-1, 2, 4, 6, 8	Wageningen Bioveterinary Research (Netherlands)	Experimental	Target species (sheep, cattle)
DISA series vaccines (replication-deficient) [24,28]	BTV-1, 2, 3, 4, 6, 8, 17, 25	Wageningen Bioveterinary Research (Netherlands)	Experimental	Target species (sheep, cattle)

**Table 2:** VP2 Antigen Epitopes of Different Serotypes.

Serotype	Epitope Sequence/Amino Acid Position	Identification of Antibodies
BTV-1 [45]	115AQPLKVGL122	4B6
BTV-1 [46]	296KEPAD300	17E9C6, 17E9C8, 17E9H12
BTV-1 [47]	619TVTAQYT 625	4A2, 4A8, 4D4, 4D5, 4E6, 4E8, 4F3
BTV-1 [47]	430RLNHSTREITYAQG 443	4B1, 4E3, 4E1
BTV-3 [48]	466EMIQSVIDDGWDQ478	2F8
BTV-3 [48]	505SQVFPSYYNKWT520	4E8
BTV-4 [49]	160NH161	4A 1G7
BTV-8 [50]	281LCRLLSTIGRKMCNTE296	2G4
BTV-8 [51]	283LL284	3G11
BTV-8 [52]	99HSEFHTKSNWVQW111	3E11
BTV-8 [52]	622AQYVFEKTCLYVLE635	4D9
BTV-13 [53] *	642EMDDDETEYE651	D24.15
BTV-15 [54]	15IAPAIIKRYP24	2D6
BTV-16 [55]	34EWSGHDVTEIPNRRMF49	3G10
BTV-16 [55]	543DPYVKRTVK551	2B4
BTV-17 [56] *	The 199th amino acid	MAb17.82, 17.83, 17.813
BTV-17 [56] *	The 213th amino acid	MAb 17.85
BTV-17 [56] *	The 327th and 582th amino acid	MAb 034
BTV-17, 10 [56] *	The 327th and 402th amino acid	034
BTV-17 [57]	540DPWNNR545	3F4
BTV-17 [57]	540DPWNNRA546	4H10
BTV-25 [58]	359LYP361	25A2B6
BTV-25 [58]	580NT581	25B2G3
BTV-4, 10, 11, 17, 20, 24, 25 [58]	620TFR622	25C4B2

Note: Asterisks (*) indicate epitopes whose neutralizing activity has been experimentally confirmed by neutralization or blocking assays.

**Table 3:** VP5 Antigenic Epitopes of Different Serotypes.

Serotype	Epitope Sequence/Amino Acid Position	Identification of Antibodies
BTV-1 [44]	144DEKQFDILNK153	4G9
BTV-1 [44]	154AVTSYNKILT163	6C9
BTV-1 [44]	207VERDGMQEEA216	3A1, 3C8
BTV-1 [44]	312ENHKELMHIK321	6B1
BTV-16 [42]	310ITANTREIQHIJKEE323	3B11, 3G9
BTV-16 [42]	265LSGID269	2B10
BTV-16 [42]	188STMVKEYRQKIDALKA203	4A6
BTV-4 [43]	119–134, 257–272, 286–301, 322–337, 481–496	multiple mAbs

Note: All VP5 epitopes identified to date are linear B-cell epitopes. None have been validated for neutralizing activity.

## Data Availability

No new data were created or analyzed in this study. Data sharing is not applicable to this article.
